# Twelve tips to facilitate the transition between research and clinical training in physician scientist education

**DOI:** 10.15694/mep.2019.000087.1

**Published:** 2019-04-15

**Authors:** Talia Swartz, Bianca Taylor-Starobin, Benjamin Chen, Margaret Baron

**Affiliations:** 1 Icahn School of Medicine at Mount Sinai

**Keywords:** physician scientist, md/phd, individual development plan, transition, clinical refresher

## Abstract

This article was migrated. The article was not marked as recommended.

Medical scientist trainees who take extended scholarly time in the middle of their training experience challenges during transitions in training. As they complete their dissertation and re-enter the clinical years of medical school, there is marked by a loss of clinical knowledge and skills over a prolonged hiatus from medicine and mismatched expectations. We have developed a re-entry pathway including guidance, support, and a course to provide clinical skills and knowledge training to align expectations, dispel myths, and allow advanced planning to meet milestones. Students reporting satisfaction with their transition from PhD to MD3 and 90% of students reported that the pathway was valuable. We provide 12 tips on how to improve the transition from research to clinical training. Here we demonstrate that a planned pathway for the transition back to medical training can improve the student-mentor experience, reduce anxiety, and create a more seamless and successful transition.

## Introduction

Taking a gap between pre-clinical training and clinical training can be a stressful experience for medical students. Such gaps occur when students take time in between phases of their medical training to pursue research or other activities. MD-PhD programs are dual degree programs that offer training in both research and medicine ( Dyrbye *et al.*, 2004). They are intended for trainees who are seeking to become physician scientists ( Nagasako, 2011; Roberts *et al.*, 2012). Most such programs structure the education such that students begin their medical training, then take a hiatus to complete their laboratory research, defend their dissertation, and then re-enter the clinical training portion of their medical education. While various permutations of this track exist, for those who take a 3-5 year gap between pre-clinical and clinical training, there exist a number of challenges in re-entering clinical training ( Smith, 2007; Whitcomb, 2007; Goldberg and Insel, 2013; School, 2013). We developed a Clinical Refresher Course in 2007 that re-acclimates students to clinical medicine through hands-on clinical exposures, extensive feedback on presentation skills, didactic pathophysiology case discussions, and peer input ( Swartz and Lin, 2014).

The Clinical Refresher Course has been well received and students report feeling a much greater level of comfort in clinical skills and greater readiness to return to clinical training. Any student who plans to complete a dissertation and re-enter medical school in the upcoming academic year is required to take this course. Despite the success of the course, we have noted that students were often unsure when they would be ready to defend their dissertation in time to rejoin the medical school class. Misalignment of goals and expectations between students and mentors results in delays and disagreements. Students experienced confusion from unclear expectations and inadequate conversation between students and mentors. The disagreements uniformly related to the lack of a continuous dialogue between students, mentors and program leadership. Our goal was to create a dialogue with students and mentors to allow them to consider progress and plans for meeting milestones in an advanced and deliberate way; that would help engage students with mentors; and that would allow for advance planning of meetings with advisory committees and appropriate preparations for dissertation defense.

To this end, we have developed a re-entry pathway in which students in the two years prior to anticipated completion of their doctoral research, along with their mentors, attend a meeting in which the process and logistics of re-entering clinical clerkships (typically the third year of medical school) is described. The goal of this meeting is to clarify expectations and help students and mentors to put together a timeline that is agreeable to all members of the students’ advising network. Focused meetings provide important milestones for completion of the PhD, necessary dates for participation in the medical school promotions process, and key dates for the Clinical Refresher Course.

The goals of the re-entry transition are:

1. To familiarize students and mentors with expectations for their final dissertation year and re-entry into the medical school curriculum.
2. To encourage dialogue between students and mentors about realistic expectations for a timeline to complete the doctoral dissertation.
3. To prevent unnecessarily lengthy dissertation timelines by identifying students who are struggling to meet milestones and encourage guidance from advisory committee members.
4. To allow a forum for addressing questions from students and mentors regarding the process.

## Tips

### 1. Create an Individual Development Plan

An Individual Development Plan is a structured tool used to develop a training roadmap to outline accomplishments and goals. It is a set of questions that allows trainees to reflect on their progress and to determine their expectations in the short-term and long term. Development of a structured IDP has been recommended by the National Institutes of Health ( NIGMS, 2018) for all trainees ( Byars-Winston *et al.*, 2011; Fuhrmann *et al.*, 2011; Vanderford *et al.*, 2018). Our MD/PhD program implemented a mandatory IDP form to be completed annually by students and reviewed with a faculty advisor / mentor. The form is intended to be educational for trainees and informative for programmatic data collection. One question identifies whether the student anticipates dissertation defense in the upcoming year, allowing the program to identify those students who are not on track to graduate in a timely manner. For any student who has been in the laboratory for more than 4 years and who indicates “no” or “uncertain”, a meeting is arranged between the student, mentor, and MD/PhD leadership to identify the reason for the uncertainty. Both student and mentor are strongly encouraged to attend a re-entry meeting that is held in September of each academic year.

### 2. Coordinate conversation with students and mentors

A meeting held in September of the year or two prior to re-entry includes all students who have been in the laboratory for more than three years, their mentors, MD/PhD program leadership, and representatives from Medical School Student Affairs. An overview of the re-entry process is outlined which emphasizes the initiation of a conversation between student and mentor regarding graduation plan and feasibility. General expectations for completion of the PhD are reviewed, including completion of experiments, committee meetings, manuscript submission, dissertation writing, defense, deposition, and vacation planning. Representatives from Medical School Student Affairs speak about the lottery process and timeline for compliance training. Students and mentors are provided with an overview of the Clinical Refresher Course and the clearly stated expectation that their time will need to be protected for 1 half day a week between April through June. A question and answer sessions allows student and mentors to explore concerns or complaints. This process has encouraged the initiation of a dialogue between students and mentors and that can help resolve conflicts and allow for discussions on realistic timelines and expectations for graduation ( Gunnarsson *et al.*, 2013).

### 3. Check in regularly

A meeting is held in January by Student Affairs for all the students who remain committed to re-entry in which they are provided with more detail about the lottery, third year clerkships, and compliance training. This meeting provides students with a clearer understanding of the milestones that must be met for the completion of the year prior to beginning clinical clerkships. Having regular check ins can be important in helping ensure that expectations and milestones are being met ( Fuhrmann *et al.*, 2011; Harrington, 2018).

### 4. Map out plans and expectations for the final graduate year

The student and mentor should discuss realistic expectations for the completion of the research, including, but not limited to experiments, publications, collaborations, committee meetings, thesis defense, revisions, and thesis submission ( Feldon *et al.*, 2010; Duke and Denicolo, 2017). It is important to set goals based on reasonable estimations of time frames and to reassess them on a regular basis.

### 5. Organize a refresher course

For students who have taken prolonged time off (more than 2 years) between phases of their clinical training, some formalized coursework is beneficial in helping them to refresh and strengthen clinical skills and reorient to the role of the third-year medical student. At our institution, students take a Clinical Refresher Course and complete their dissertation. Their time is protected in the laboratory for this clinical exposure and mentors understand the demands made on the students during this challenging time. The course involves one full day orientation followed by 1 half-day per week, for 8 weeks. Other details have been previously described ( Bills *et al.*, 2013; Swartz and Lin, 2014).

### 6. Prioritize wellness. Plan vacation time

Students begin their MD3 year in the last week of June, just prior to the start of clerkships, with a week of intense clinical skills training. They enter clerkships in July. It is emphasized that they should plan ahead to be sure that they have time for a well-deserved vacation after the completion of their dissertation work and the Clinical Refresher Course. It is especially importance for these students to prioritize wellness, as this is a critical time during training when multiple high-stakes responsibilities coincide and the unknown of the future phase of training is a source of anxiety ( Lyndon *et al.*, 2017a; Lyndon *et al.*, 2017b; Ziegelstein, 2017; Erschens *et al.*, 2018; Pathipati and Cassel, 2018). It is important for students to know that they are not alone and that they can and should take care of their physical and mental health.

### 7. Pair students with peer advisors

Student peer advisors are an invaluable resource ( Moore-West *et al.*, 1990; Andre *et al.*, 2017). They have experienced the unique challenges posed to this cohort of students and can advise on the best study tools and approaches to take during their transition year. In our program, a panel of Student Advisors was identified based on a self-selected cohort of students in their final year of clinical training. They are instrumental in meeting with students before the return to clinical training, providing them with suggestions and developing electronic resources to help in clerkship and elective planning ( Gunn *et al.*, 2018).

### 8. Pair students with faculty advisors

Students are assigned to faculty advisors who understand the complexities of clinical clerkships. Ideally that advisor would also understand the unique challenges posed to MD/PhD students making the transition back to medical school in the midst of completing a dissertation. This advisor can help to allay anxieties, plan appropriately for any needed flexibility in the transition, and help reinforce important milestone planning ( Aagaard, 2015; McLuckie *et al.*, 2018).

### 9. Expose students to clinical clerkships so that they know what to expect

The unknown of clerkships can be daunting to students, particularly those who have taken a prolonged gap between preclinical and clinical training. Introducing a preview into clerkships and expectations can help to allay some of these anxieties and give students a sense of the life of a third year clerk before they begin ( Sheu *et al.*, 2018). We have introduced a morning session in which students are allowed to shadow an internal medicine resident-run team on the wards. MD/PhD students report variable responses to how well the team was run, but uniformly reported that the experience was enlightening and gave them a better picture of how clinical clerkships are structured.

### 10. Embrace diversity

Dissertations are not meant to be uniform. The goal of the PhD is to develop a novel hypothesis and to obtain evidence to support your new ideas. Therefore, the timeline may not and should not conform to a preset time. Multiple efforts at the institutional level can help to reduce the time to graduation, e.g. implementing an IDP, requiring regular (annual or biannual) advisory committee meetings, frontloading and overlapping required courses with medical school requirements. Despite these interventions, all students may not complete their doctoral training within the same time frame and, therefore, it is important to build in some flexibility with respect to the re-entry pathway. For example, research work might be continued during elective time built into a clerkship. Another possibility might be to defer a required clerkship to the fourth year, allowing the student to enter within the same academic year and graduate at the same as those classmates who entered at the start of the year.

### 11. Celebrate achievement

PhD theses can vary in length and the associated work may be published broadly or in highly specialized journals. Regardless, it is important to celebrate the achievement of students as they transition from one phase of training to the next. The completion of a doctoral dissertation is a formidable accomplishment and each student’s achievements should be celebrated as they master a scientific discipline and transition into clinical training. This transition marks an important one in which they will experience a steep learning curve needing to acquire new skills while not using many of those they have spent the past several years cultivating. A celebration can mark this transition together with students and faculty and can boost morale and help students feel proud of their accomplishments.

### 12. Reflect, evaluate processes, and modify as needed

Students are asked to evaluate their transition experience during an intersession week between the first and second blocks of clerkships in September following their re-entry. The survey asked students to evaluate their satisfaction with the overall transition experience, advising during the transition, the quality of the Clinical Refresher Course in preparing them for MD3, and whether the planning meeting the prior year was valuable and/or informative. They rated their transition experience from PhD to MD3 (4.1, Likert 1-5), the quality of advising leading up to MD3 (4.2, Likert), and the quality of the Clinical Refresher course in preparing them for MD3 (4.5, Likert 1-5). 90% of students reported that the planning meeting with mentors and students the year prior was valuable and 90% reported that the meeting was informative. Responses to the Clinical Refresher Course included sentiments that it helped to ease the transition back to medical school, it was appropriately tailored, and it helped relieve anxiety associated with the transition. The responses to the question “What challenges do you face in MD3?” overwhelmingly pointed to a lack of preclinical knowledge. In response to the question: “What advantages do you have as an MD/PhD student in MD3?” students cited feeling more mature than their medical student colleagues and better able to navigate the unknown and having a greater comfort level with analysis of data and practice of evidence-based medicine.

## Conclusions

Overall, the program has fundamentally changed the way that MD/PhD students approach the transition from PhD years to MD3 training. In the past, students reported great anxiety in approaching the transition, citing the unknown, a lack of preparedness, unclear expectation, and unwelcome discussions with their mentors. Through developing this course and this timeline that forces conversations with mentors well in advance to help students anticipate expectations and be able to meet milestones in a timely fashion, students are reporting feeling more satisfied with their overall transition experience (4.1, Likert 1-5), their advising during the transition (4.2, Likert 1-5), and the quality of the Clinical Refresher Course in preparing them for MD3 (4.5, Likert 1-5). 90% of students found the planning meeting in September valuable and informative. We plan to continue this program to optimize student preparedness and student-mentor communication. This program improves dialogue regarding graduation planning and increases student satisfaction as they navigate the challenging transitions in the program.

Transitions in training are particularly challenging for MD/PhD students, who experience prolonged gaps in their training and need to recover large skill and knowledge sets. It will be of great interest to evaluate the long-term effectiveness of this pathway. Outcomes to consider would include student performance in their clerkships, whether the program improves their exam scores and residency match, and whether this pathway is effective at reducing time-to-degree.

One major concern is that students who take a prolonged gap between preclinical and clinical training will always experience a knowledge gap. While courses and intense review may help to close this gap, the reality is that some knowledge will not be recovered unless it is specifically needed. Students can be reassured that much of their clinical learning will build off of fundamentals that they can review as needed, and that an important skill moving forward will be to understand what knowledge needs to be reviewed in order to meet the clinical needs at hand.

Here we demonstrate that development of a pathway for re-entry of MD/PhD students from graduate training to medical training can improve the student and mentor experience and reduce the anxiety surrounding re-entry. Our observations indicate that encouragement of dialogue between students, mentors, and MD/PhD, medical and graduate school leadership can promote a more seamless transition from graduate training back to medical school training. The success of the program depends on open communication of all parties and the ability to identify concerns by milestone tracking and conflict resolution to ensure that problems are resolved in an expedient fashion. The goal will be to continue to develop this pathway along with rigorous evaluative measures to determine the extent to which students and mentors benefit from these interventions.

## Take Home Messages

• A re-entry pathway for students returning to clinical medicine from prolonged research should include guidance and clinical skills training
• Individual development plans help to align goals between students, mentors, and program leadership
• Multiple avenues of communication can improve outcomes and program satisfaction

## Notes On Contributors

Talia H. Swartz, MD, PhD https://orcid.org/0000-0003-1572-045X is Assistant Professor in the Division of Infectious Diseases at the Icahn School of Medicine at Mount Sinai and is Associate Director of the Medical Scientist Training Program. She co-directs the Clinical Refresher Program at the Icahn School of Medicine at Mount Sinai, oversees the transition from graduate training to clinical training, and serves as Faculty Advisor for the MD/PhD students in the program. Her clinical interest is in HIV and her research interests include HIV pathogenesis and inflammation.

Bianca Taylor-Starobin, MSEd is Program Manager of the Medical Scientist Training Program. She oversees the communications and transitions of students and mentors and coordinates the meetings and communication strategies related to milestones and student information dissemination.

Benjamin K. Chen, MD, PhD https://orcid.org/0000-0002-5404-1997 is Professor in the Division of Infectious Diseases at the Icahn School of Medicine at Mount Sinai and is Associate Director of the Medical Scientist Training Program. He co-directs the first year core course for MD/PhD education in biomedical sciences and oversees preclinical advising and laboratory research for MD/PhD students in their research phase of training. Dr. Chen is Vice Chair of Research in the Department of Medicine. His research interest is on mechanisms of HIV transmission between cells.

Margaret H. Baron, MD, PhD https://orcid.org/0000-0003-3566-570X is Professor of Medicine in the Division of Hematology and Medical Oncology and Senior Associate Dean and Director of the MD-PhD Program at the Icahn School of Medicine. Dr. Baron oversees the advising and transitions of students between research and clinical training and coordinates with the MD-PhD program leadership to coordinate high quality training in biomedical sciences. Her research interest is on the regulation of erythropoiesis.
