# Supplementary material for: Twelve tips to facilitate the transition between research and clinical training in physician scientist education
Source: MedEdPublish (2016). 2019 Jun 27;8:87. Originally published 2019 Apr 15. [Version 2] doi: 10.15694/mep.2019.000087.2 (PMC10712640; doi:10.15694/mep.2019.000087.2)
Supplement: Peer review history

# Reviewer report: Reviewer response for version 2

**DOI:** 10.21956/mep.20244.r31393

**Published:** 2021-07-18

**Authors:** Helena Filipe

**Affiliations:** 1 Hospital of the Armed Forces/PL-EMGFA

This review has been migrated. The reviewer awarded 4 stars out of 5 Thank you for the opportunity to review this article. Despite the availability of programs offering a dual degree, the word “transitioning” shows the challenges to combine / merge both ends for the best outcomes of those interested. Usually rich of opportunities and new options, interface zones present the challenges of the uncertainty of the next one and the need to conclude the former to be able to fully embrace the future. Limbal zones can offer exciting growing opportunities, and guidance will be more than adequate at this stage.In an interesting manner, the Authors intertwine the tips in how best to leave one stage to embrace the next one with a particular focus on concluding the PhD phase. By reading the article, one could say that these hybrid programs, besides offering the twofold opportunity of learning to be PhD-MD professionals, the participants benefit of guidance on how to conclude their PhD program more effectively. This is particularly shown in tip 12. Probably the questionnaire that scaffolds this same tip could be worked in a different article by the Authors and create solid evidence. Besides highlighting the experience of the Authors, most of the tips show to be in accordance to the literature reviewed.This article will interest curriculum developers, scientists-mentors, clinical educators and in some extent future PhD-MDs.

Reviewer Expertise:

NA

No decision status is available

# Reviewer report: Reviewer response for version 2

**DOI:** 10.21956/mep.20244.r31392

**Published:** 2020-05-13

**Authors:** Richard Hays

**Affiliations:** 1 James Cook University

This review has been migrated. The reviewer awarded 3 stars out of 5 This is a review of Version 2. I did not formally review Version 1 but have followed the discussion thread and agree with the comment that some concerns were not addressed. I think the topic is important and rather complicated. Students in dual pathway programs have told me that it is rather like being in two races at once. Both races are in a similar direction but there are different hurdles to jump, some unexpected. Research and professional clinical training are very different. Research lab work is even more different. It may be easier to step completely out of the clinical track and focus on research, but this may make more difficult the return to clinical training. The authors focus on MD/PhD programs but my experience at other levels suggests that similar issue prevail. I found the 12 steps interesting, although rather brief. Perhaps examples for some would help elaborate the issues and approaches to their resolution. I also wonder if two other issues could be emphasised more strongly. The first is a need at the start for clear, agreed objectives, timelines, milestones and opportunities for re-negotiation. Supervisors for both tracks should meet regularly with the student. This is there, but spread out over several points. The second is a need for a dispute resolution process if things do not work out as intended. Combined research/clinical pathway programs are not as popular as many expected, as they are very hard work, although some institutions have been successful. Understanding differences between more and less successful programs would be helpful. The paper should be of interest to all involved in combined pathway programs.

Reviewer Expertise:

NA

No decision status is available

# Reviewer report: Reviewer response for version 2

**DOI:** 10.21956/mep.20244.r31391

**Published:** 2019-07-02

**Authors:** Ken Masters

**Affiliations:** 1 Sultan Qaboos University

This review has been migrated. The reviewer awarded 3 stars out of 5 This is a revised version of the paper, in which the authors have attempted to address the concerns raised about the original version.Unfortunately, the authors have not adequately addressed all of the important points.• In an effort to clarify the time line of events, the authors have made small changes, such as changing “September” to “Fall” and changing “January” to “Winter”; unfortunately, this does not really help much. In my comments on the original version, I pointed out that the timing should give “some idea of a number of months before the start of the academic year, and or relationship between these timings and vacations/holidays, etc”. The reason was that “academic years begin at different times of the calendar year for different institutions and countries”. For instance, in many countries in the southern hemisphere, the academic year more closely resembles the calendar year, with the year starting in January/February/March. So, really, what we are looking for is something like “One month before the start of the academic year,” or “At the beginning of the academic year,” or “one month before re-entry,” etc. Unfortunately, only changing months to seasons does not address this problem. (In addition, there are places (e.g. “between April through June”) where no changes have been made. Although the reader might then be able to tie this to the later reference to the start of the clerkship in July, that is indicated only 4 tips later, so the original month designation is uncertain in the timeline context of other events). • Tip 12 had the problem that the percentages cited had no real context, and “numbers of students, demographics, dates, number of times the survey was run, etc. are required” (or, if the information were available elsewhere, then those sources should be cited). Unfortunately, in the revised version, while parts of Tip 12 have been re-written, this information has not been supplied nor cited. The Tip still speaks of “The survey” with no information about the survey, how it was run, or numbers of subjects, etc. (In their response to the comment on the previous version, the authors did indicate that they were emphasising the trend, but, in this revised version, they still indicate a figure of 90% of the students “reported that a planning meeting with mentors and students the year prior was valuable and informative” without giving a suitable context for this 90%).• Smaller issue: although the authors have placed the abbreviation IDP after the use of the term “Individual Development Plan”, it really should have been done after the first instance of the use of the term (in line 1 of Tip 1). So, although there have been some changes to the paper, unfortunately, the revision still suffers from the main problems of the first version: an unclear timeline, and uncontextualised statistics.

Reviewer Expertise:

NA

No decision status is available

# Reviewer report: Reviewer response for version 1

**DOI:** 10.21956/mep.18992.r27231

**Published:** 2019-05-22

**Authors:** Ken Masters

**Affiliations:** 1 Sultan Qaboos University

This review has been migrated. The reviewer awarded 3 stars out of 5 The paper gives tips on how to facilitate the transition between research and clinical training in physician scientist education; this problem occurs when students divert from their clinical training into academic work, and then return to their clinical training.The authors have grounded the introduction to their paper well in the literature, and then draw their advice based upon the Clinical Refresher Course that they have developed as a “re-entry pathway”. All the of tips are well-grounded in both experience and literature.The problem is that the tips are based so heavily on a single course at a single institution, and the paper seems to waver between a report of an individual course survey and a generalisation of the experience into advice, but the generalisation is not broad enough: • Some of the ideas expressed are directly related to a narrow context, and should be generalised more. For example, “A meeting held in September of the year or two prior to re-entry” and “A meeting is held in January by Student Affairs” (and similar references elsewhere) make sense only if one knows exactly when the academic year begins. As academic years begin at different times of the calendar year for different institutions and countries, it would be better if the authors gave some idea of a number of months before the start of the academic year, and or relationship between these timings and vacations/holidays, etc.• Similarly, “frontloading” is not a universally common educational term, and should be explained.• Tip 12 introduces statistics from a survey of the course. For these statistics to have validity, the authors really do need to supply more information. I am not suggesting a full-blown research report, but numbers of students, demographics, dates, number of times the survey was run, etc. are required. Either that, or, if the evaluation has been published elsewhere, then it is necessary to cite it. Otherwise, there are really just vague numbers and percentages.• The conclusion appears to repeat these statistics (although the phrasing of the surrounding text is a little different, requiring the reader to refer again to Tip 12 to confirm that these are same statistics.)• In fact, the conclusion appears to be more fitting to the conclusion of a research paper dealing with an evaluation of a course; it is almost as if the authors originally wrote it as an evaluation of a course, and then converted it into a 12-tips paper. I’m not sure if that is the case, but that is the impression that is given. So, I would recommend that the authors re-look at their conclusion, and focus on it as a conclusion to a series of tips.Small issues:• The abbreviation IDP should be inserted in brackets immediately after the term Individual Development Plan is used.Overall, the paper has the kernel of a useful solution to a (albeit not common) problem, but the authors need to ensure that they generalise their advice far more and supply required details behind their statistics in order for it to be of value to all other readers.

Reviewer Expertise:

NA

No decision status is available
